# Comprehensive dataset of annotated rice panicle image from Bangladesh

**DOI:** 10.1016/j.dib.2023.109772

**Published:** 2023-11-04

**Authors:** Mohammad Rifat Ahmmad Rashid, Md. Shafayat Hossain, MD Fahim, Md. Shajibul Islam, Tahzib-E-Alindo, Rizvee Hassan Prito, Md. Shahadat Anik Sheikh, Md Sawkat Ali, Mahamudul Hasan, Maheen Islam

**Affiliations:** Department of Computer Science and Engineering, East West University, Dhaka, Bangladesh

**Keywords:** Object detection, Rice panicle, Annotated image, Crop yield estimation, Computer vision

## Abstract

Bangladesh's economy is primarily driven by the agriculture sector. Rice is one of the staple food of Bangladesh. The count of panicles per unit area serves as a widely used indicator for estimating rice yield, facilitating breeding efforts, and conducting phenotypic analysis. By calculating the number of panicles within a given area, researchers and farmers can assess crop density, plant health, and prospective production. The conventional method of estimating rice yields in Bangladesh is time-consuming, inaccurate, and inefficient. To address the challenge of detecting rice panicles, this article provides a comprehensive dataset of annotated rice panicle images from Bangladesh. Data collection was done by a drone equipped with a 4 K resolution camera, and it took place on April 25, 2023, in Bonkhoria Gazipur, Bangladesh. During the day, the drone captured the rice field from various heights and perspectives. After employing various image processing techniques for curation and annotation, the dataset was generated using images extracted from drone video clips, which were then annotated with information regarding rice panicles. The dataset is the largest publicly accessible collection of rice panicle images from Bangladesh, consisting of 2193 original images and 5701 augmented images.

Specifications Table

**Table:** 

Subject	Agriculture and biological science
Specific subject area	Computer vision techniques for the detection of rice panicle head for crop yield estimation.
Data format	Raw, and analyzed
Type of data	Image, and JSON file
Data collection	On April 25th, 2023, data collection took place in Bonkhoria, Gazipur, Dhaka, Bangladesh, from 9:00 AM to 2:00 PM for a total of 5 h. Data was collected using a DJI Mavic Mini 2 Drone for aerial surveillance and photography. The drone's camera captured high-quality images and videos. The collected data was recorded in MP4 format with a video resolution of 4 K (3840×2160 pixels) at 30 frames per second, allowing for precise and vibrant footage. Multiple frames were extracted from these video footages, and these frames were used for the annotation process. The images were annotated using the Roboflow platform [1] image annotation tool. Finally, a total of 2193 original rice panicle images were created, and after applying seven types of augmentation, this count increased to 5701 images.
Data source location	• Institution: Cultivated rice field of size 130,680 square feet by a local farmer. • City/Town/Region: Bonkhoria, Gazipur • Country: Bangladesh • Latitude and longitude: 24°03′55.6″N 90°25′42.9″E
Data accessibility	• Repository name: Mendeley Data • Data identification number: 10.17632/ndb6t28xbk.4 • Direct URL to data: https://data.mendeley.com/datasets/ndb6t28xbk/4

## 1 Value of the Data

• Traditional techniques for estimating rice output in Bangladesh require a long duration and are unreliable. Rice production may be precisely estimated using deep learning models trained on this dataset, which can assist farmers in making better crop management decisions. The dataset, for instance, may be used by a researcher to build a deep-learning model that can automatically find and count rice panicles in drone photos [2,3]. When applied to estimate rice production in real-time, a computer vision-based approach can assist farmers in making better crop management decisions [4].
• Analyzing phenotypes can be aided by this dataset. The analysis of phenotypes is the study of an organism's or plant's observable traits [2,5,6]. The association between rice panicle density and other plant traits like yield, growth rate, and disease resistance [7] may be better understood with the use of this dataset, which can be utilized by researchers. This information, for instance, might be used by a researcher to examine how different fertilizer applications affect the density of rice panicles. The researcher might then apply this knowledge to suggest the appropriate fertilizer application for a certain crop.
• This dataset can be used to create new agricultural technology. These technologies can aid farmers in increasing agricultural yields and lowering production expenses [8,9]. Using this information, for instance, drones with autonomous rice panicle detection and counting capabilities may be created. Farmers may then examine their fields with the help of these drones to spot regions with poor agricultural yields. In order to increase agricultural yields, this information may subsequently be utilized to target these regions with irrigation or fertilizer.

## 2 Data Description

**Fig. 1:**
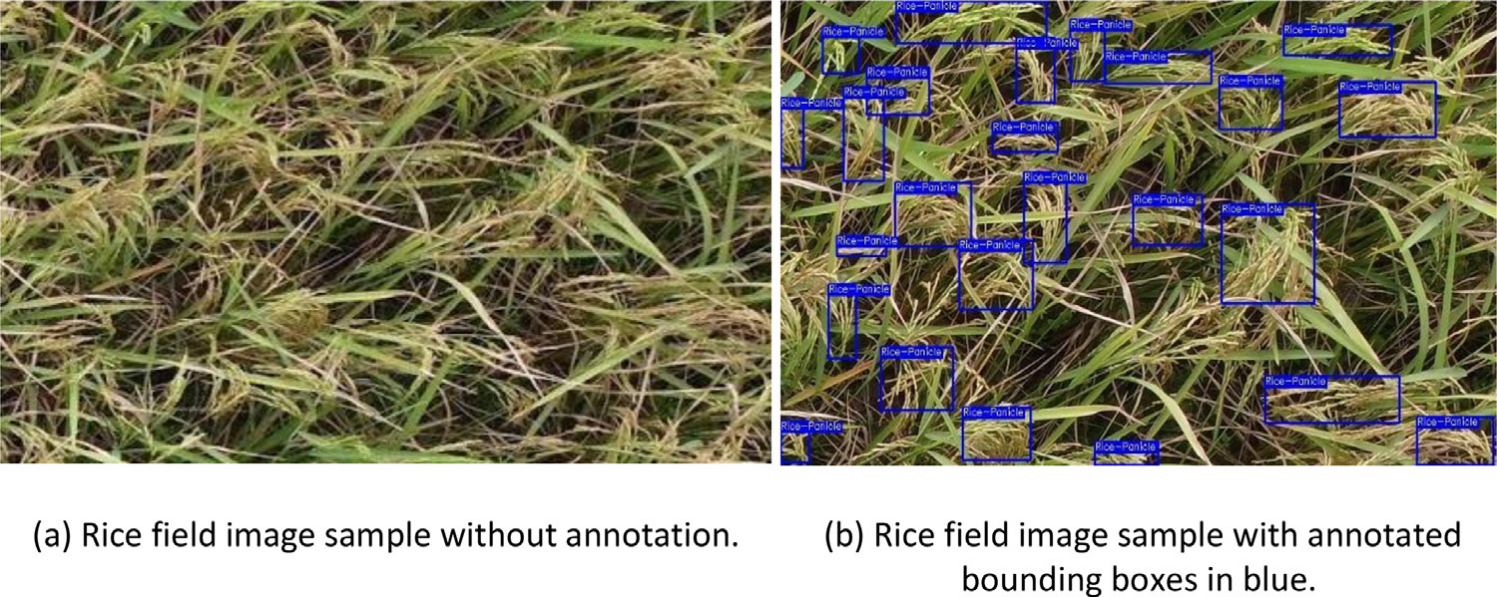
Rice field image samples: (a) image without annotated bounding boxes and (b) image sample with annotated bounding boxes in blue. (For interpretation of the references to color in this figure legend, the reader is referred to the web version of this article.)

We collected 2193 original rice field images from the drone video footage. These images were annotated by applying bounding boxes around the rice panicles, utilizing the Roboflow annotation Tool [1]. We employed a single class label for annotation, which is labeled as “rice-panicle”. An example of an annotated rice panicle using bounding boxes is shown in Fig. 1. All images are encoded in the standard JPG format, while the generated annotations are presented in the COCO format [10] within a JSON file. In this format, the bounding box is represented by four values: the x and y coordinates of the top-left corner and the width and height of the box. These values are all normalized, representing them as fractions of the image width and height.

We perform seven types of augmentation techniques such as flip, rotate, mosaic, hue, saturation, brightness, and exposure on the dataset, thereby producing an extended version of the original dataset, tailored for various deep learning tasks. As we collected data from the experimental setup, we applied augmentation to diversify and enrich the dataset, simulating real-world scenarios. Augmented data introduces variations that mimic practical conditions, enhancing the robustness and generalization of deep learning models. It enables models to adapt to various scenarios, including changes in lighting, orientation, or perspective frequently encountered in practical applications. To achieve this, we employed Flip, Rotate, and Mosaic Augmentation techniques [11].

Additionally, augmented data serves to simulate real-world environments and conditions. Researchers can assess model robustness and reliability under different environmental factors, such as lighting changes or image distortions. To account for environmental variations, we applied Hue, Saturation, Brightness, and Exposure adjustments [12]. This augmentation process resulted in the creation of 5701 rice panicle images, occupying approximately 851 MB of storage.

**Fig. 2:**
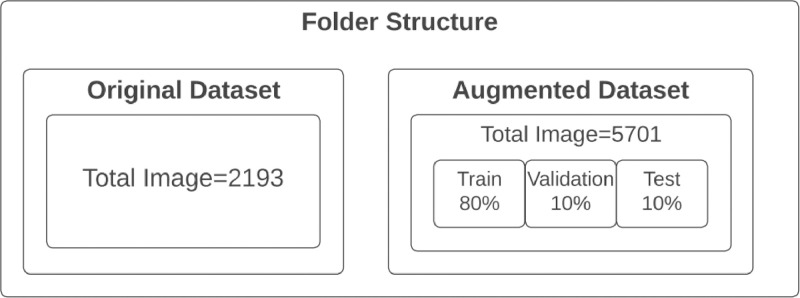
Folder structure of the repository.

These images are now well-prepared for deployment in training machine-learning models that target the detection of rice panicles. Furthermore, we partitioned the augmented dataset into three subsets: training (80 %), validation (10 %), and test (10 %). The folder structure of the dataset repository is illustrated in Fig. 2. In order to maintain an organized structure, we divided the dataset repository into two main sections. The first folder consists of the original images along with their corresponding annotation file in JSON format, compressed within a zip file containing 2193 images. The second folder has the augmented images, accompanied by their annotation JSON file, all contained within a zip file encompassing the 5701 augmented images.

## 3 Experimental Design, Materials and Methods

**Fig. 3:**
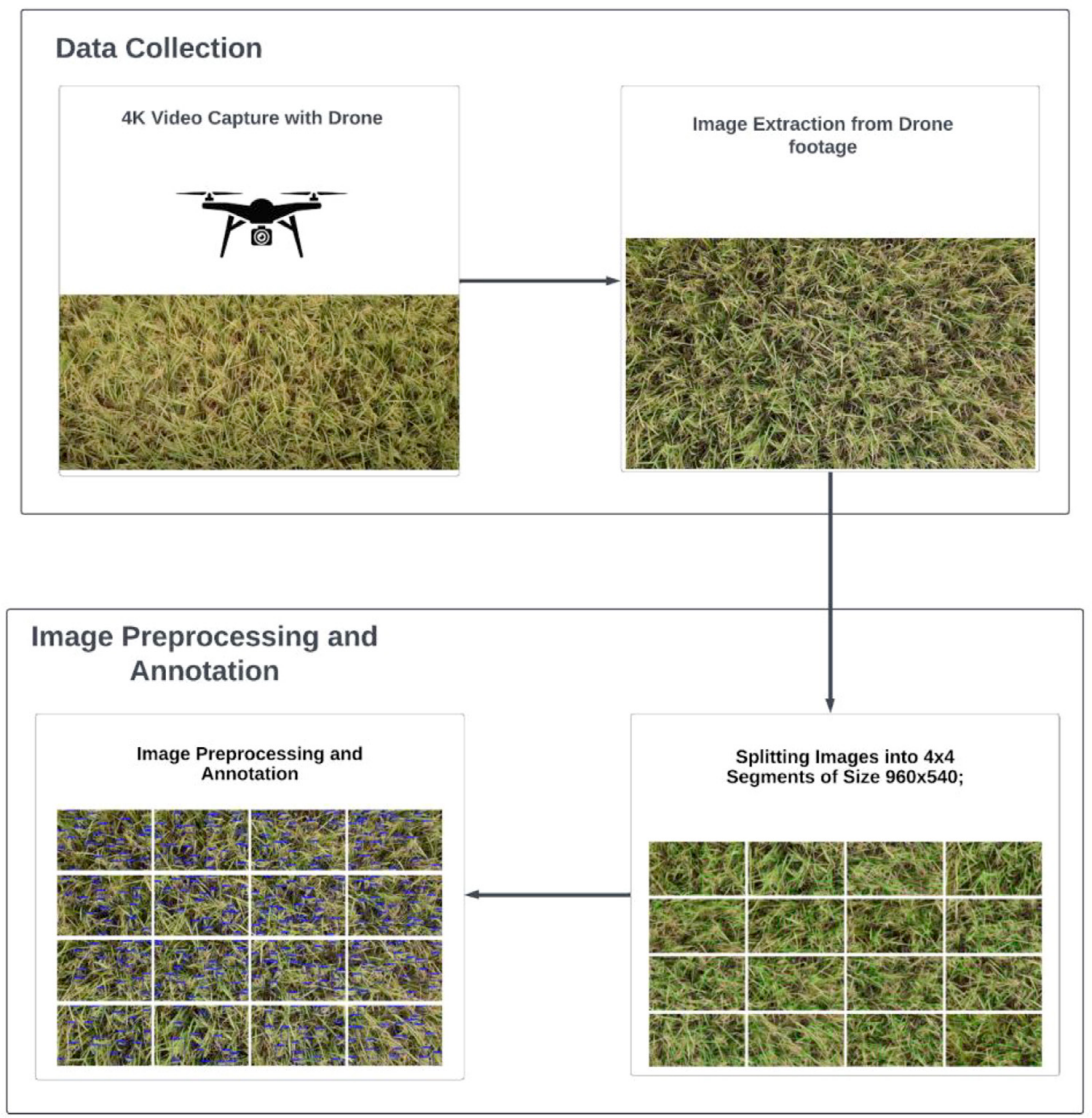
High-level overview of the experimental setup for rice panicle data collection and creating an annotated image by splitting the extracting image into a 4 × 4 image size of 960×540.

Fig. 3 presents a high-level overview of the experimental setup for rice panicle data creation from the rice field. The four main steps of our experimental setup are (a) 4 K Video Capture with a Drone; (b) Image Extraction from drone footage; (c) Splitting Images into 4 × 4 Segments of Size 960 × 540; and (d) Image Preprocessing and Annotation. The drone flies over the rice field, capturing 4 K video footage of the rice panicles. For image acquisition, the DJI Mavic Mini 2 Drone captures aerial footage. The drone is equipped with a 1/2.3″ CMOS sensor and a 12-megapixel resolution. With an aperture of f/2.8 and a field of view (FOV) of 83°, the drone captured high-resolution video of wide surroundings. The drone has a built-in image stabilization capability for smoother video footage, allowing us to extract frames without blurriness. The process of image extraction, image preprocessing to 4 × 4 splitting, and image annotation was done in the Roboflow platform [1].

### 3.1 Data collection pipeline

**Fig. 4:**
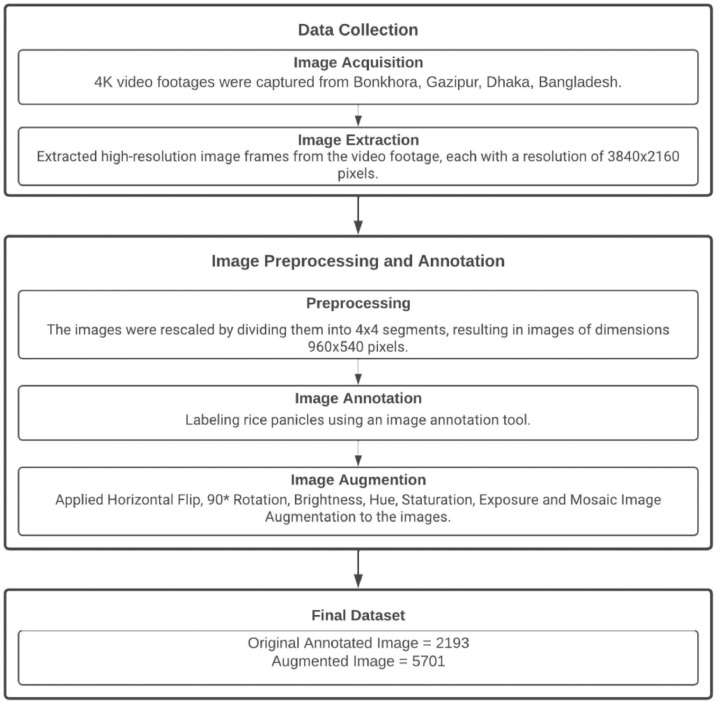
Complete data collection pipeline which consists of image acquisition, image extraction, preprocessing, image annotation, and image augmentation.

Fig.4 illustrates the complete data collection pipeline which consists of image acquisition, image extraction, preprocessing, image annotation, and image augmentation. It is divided into two parts: (1) Data Collection, and (2) Image Preprocessing and Annotation. The data collection stage contains two steps, image acquisition and extraction. The first step is about acquiring the video footage to create the dataset and the second is about extracting the data frames from the video for the rice panicle annotation process. We collected all videos at a height of 2.07 m from the rice field, with only one video captured at a height of 3.5 m from the rice field.

**Fig. 5:**
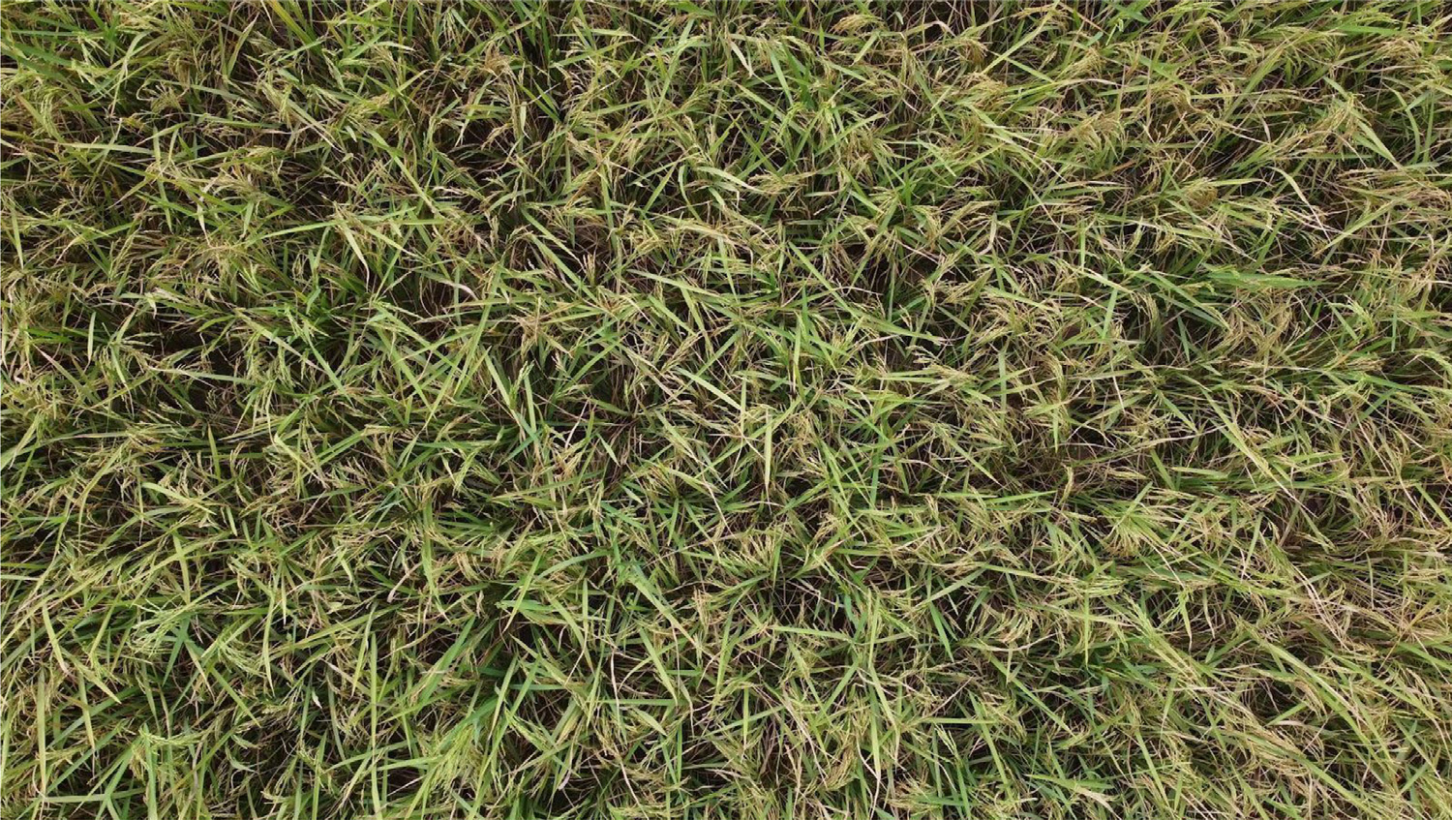
4 K resolution image from rice field video footage.

**Fig. 6:**
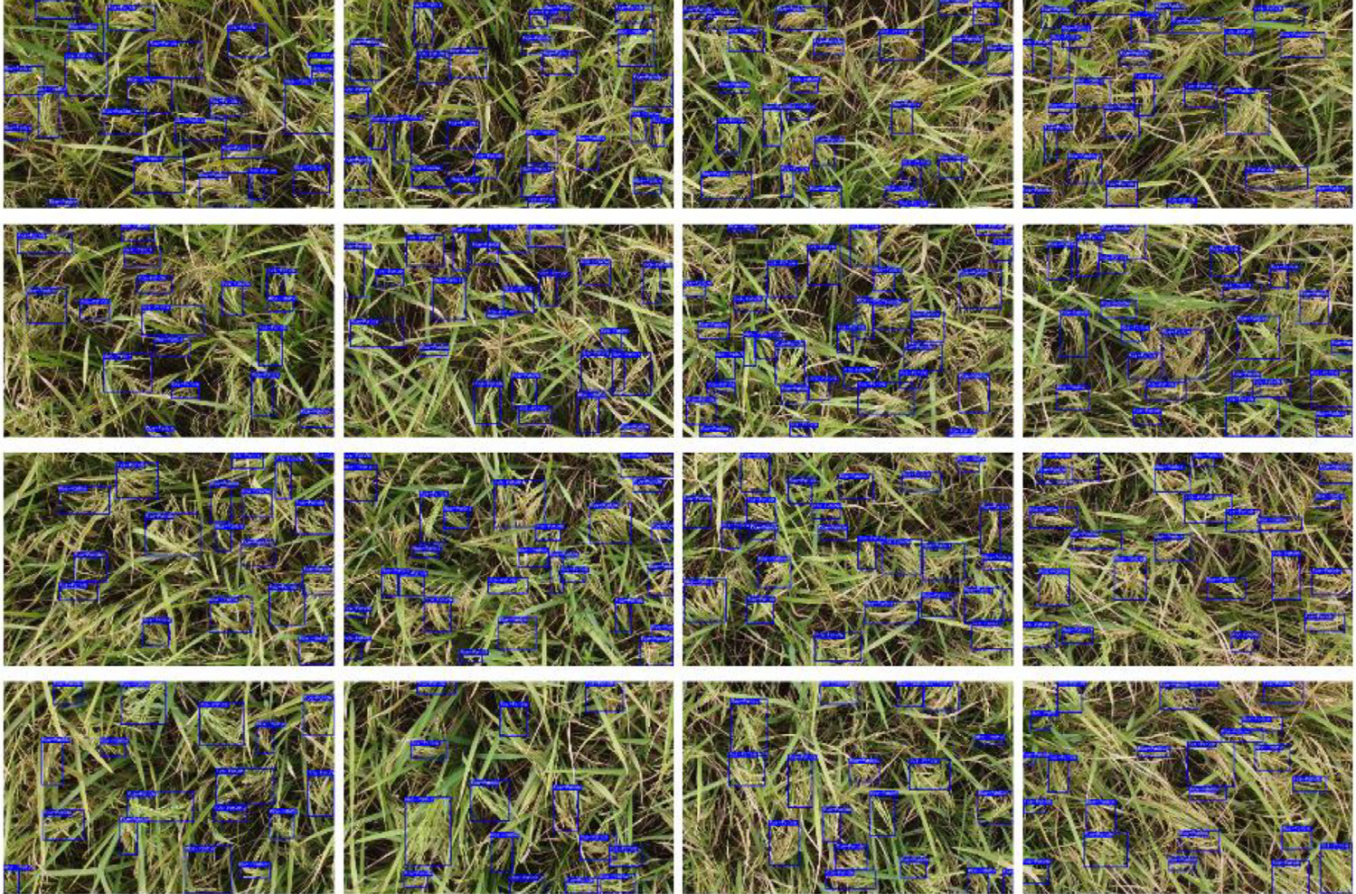
Rescaling the high-resolution image into 4 × 4 image segments of resolution 960×540 pixels and rice panicle annotation bounding boxes in blue color.

Data collection for the rice panicle images took place in Bonkhoria, Gazipur, Dhaka, Bangladesh, on April 25th, 2023. The data was collected during daylight hours under the open sun, ensuring optimal lighting conditions. We captured the dataset using a high-end DJI Mavic Mini 2 Drone with the assistance of an expert drone operator for precise control. The primary data collected was in video MP4 format. A frame from the video file is shown in Fig. 5. We obtained a total of five video footages, collectively amounting to 7 min and 52 s. These five footages were captured from various angles and perspectives to maintain the flexibility of our dataset. Multiple images were then extracted from these raw video files. Next, we divided the 4 K resolution image into smaller segments, creating 4 × 4 image segments with a resolution of 960×540 pixels, as illustrated in Fig. 6.

The second stage of the data collection process focuses on image annotation, and it is divided into three phases. In the first phase, images were rescaled by partitioning them into 4 × 4 segments, resulting in images with dimensions of 960×540 pixels. In the second phase, the image annotation process involved labeling rice panicles using the image annotation tool provided by the Roboflow platform [1]. An annotated rice panicle image with bounding boxes is shown in Fig. 6. After the image augmentation process, we created the final dataset. In the final stage, we have further filtered the newly annotated images by removing anomalies, and errored images, and removing images that have bounding box areas that are less than the mean of all the bounding boxes area.

### 3.2 Image preprocessing and annotation

We applied a variety of preprocessing and augmentation techniques to enhance the dataset. Initially, the images were resized to a standardized dimension of 960×540 pixels. Our augmentation strategy encompassed combining various transformations that help to diversify and enrich the dataset, simulating real-world scenarios. We include the following seven augment techniques.

• Flip (Horizontal) [12]: This technique flips the image horizontally, creating a new image that is the mirror image of the original image. This can help to increase the diversity of the dataset and make the model more robust to changes in perspective.
• 90° Rotation [12]: This technique rotates the image by 90° in either a clockwise or counterclockwise direction. This can also help to increase the diversity of the dataset and make the model more robust to changes in perspective.
• Hue (between −25° and +25°) [12]: This technique adjusts the hue of the image, which is the color of the light reflected by the object in the image. This can help to increase the diversity of the dataset and make the model more robust to changes in lighting conditions.
• Saturation (between −25 % and +25 %) [12]: This technique adjusts the saturation of the image, which is the intensity of the color in the image. This can help to increase the diversity of the dataset and make the model more robust to changes in lighting conditions.
• Brightness (between −25 % and +25 %) [12]: This technique adjusts the brightness of the image, which is the overall lightness or darkness of the image. This can help to increase the diversity of the dataset and make the model more robust to changes in lighting conditions.
• Exposure (between −5 % and +5 %) [12]: This technique adjusts the exposure of the image, which is the amount of light that is captured by the camera. This can help to increase the diversity of the dataset and make the model more robust to changes in lighting conditions.
• Mosaic Augmentation [11]: This technique creates a new image by combining four randomly selected images from the dataset. This can help to increase the diversity of the dataset and make the model more robust to occlusion and other challenging conditions.

In the augmentation process, flipping the images can help make the model more robust to change in perspective, such as when viewing rice panicles from different angles. Rotating the images can also enhance the model's robustness to changes in perspective. Adjusting the hue, saturation, brightness, and exposure of the images can improve the model's ability to handle changes in lighting conditions. Mosaic augmentation can enhance the model's robustness to occlusion and other challenging conditions [11]. It is a powerful and versatile data augmentation technique that can be used to improve the performance of computer vision models on a variety of tasks. It is particularly important for computer vision datasets because it can help to address a number of common challenges, such as object localization, context, small objects, and data imbalance. In the mosaic augmentation procedure, the first step is to randomly select four images from the training dataset. Once four images have been selected, each image is resized to a random size. This helps to create more diverse augmented images, and it also helps to ensure that the objects in the augmented images are of different sizes. The next step is to stitch the four resized images together into a new image. Once the four images have been stitched together, the bounding boxes for the objects in the four original images need to be transformed and added to the new image. The final step is to take a random crop from the new image. This helps to create even more diverse augmented images, and it also helps to ensure that the objects in the augmented images are in different locations.

**Fig. 7:**
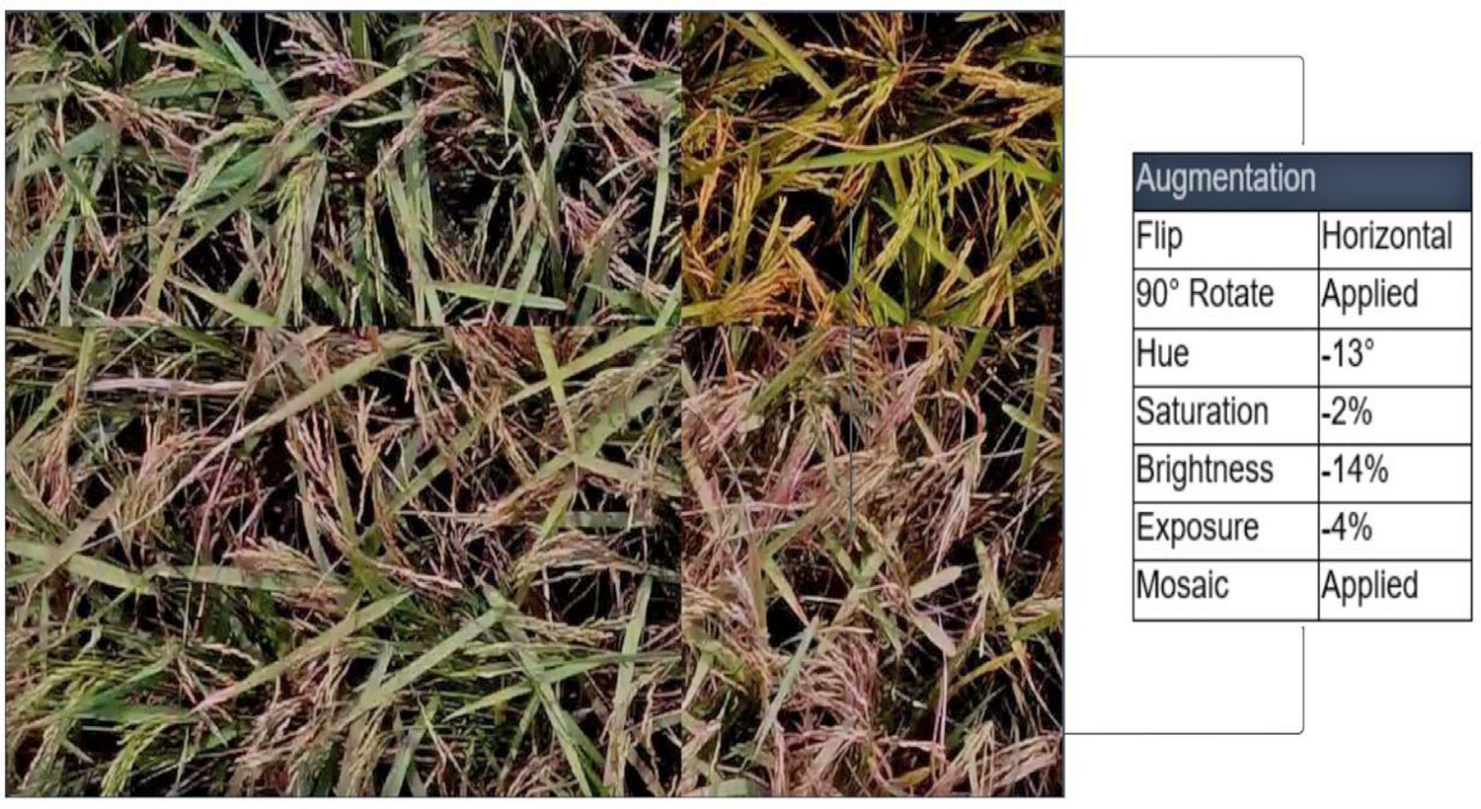
Augmented image with horizontal flip, 90° rotation, −13° hue, −2 % saturation, −14 % brightness, −4 % exposure and image with applied mosaic augmentation.

After applying all seven augmentations to the original dataset, it increases the dataset size by approximately 1.6 times the original dataset. Fig. 7 illustrates an augmented image with horizontal flip, 90° rotation, −13° hue, −2 % saturation, −14 % brightness, −4 % exposure, and an image with applied mosaic augmentation. During the image annotation process, we used the YOLOv8 [6] model, which we trained on the Roboflow platform [13] utilizing the preprocessed and augmented dataset. This enabled us to automate the detection of rice panicle heads during the manual annotation process. Furthermore, we structured the annotation procedure in a semi-supervised manner. This strategic approach offers an additional advantage by paving the way for potential dataset expansion. For instance, the pre-trained model could be effectively used for the automatic annotation of new rice field images, followed by manual refinement to ensure the accuracy of the labeled images.

## Ethics Statement

The authors adhere to the journal's ethical guidelines and confirm that this research does not involve humans, animals, or data obtained from social media. The datasets utilized in the study are publicly accessible, and appropriate citation protocols should be followed when utilizing these datasets.

## CRediT authorship contribution statement

**Mohammad Rifat Ahmmad Rashid:** Supervision, Writing – review & editing, Conceptualization. **Md. Shafayat Hossain:** Methodology, Software, Formal analysis, Data curation, Validation. **MD Fahim:** Data curation, Investigation, Writing – original draft, Resources. **Md. Shajibul Islam:** Project administration, Writing – original draft, Writing – review & editing, Data curation. **:** Visualization, Data curation, Writing – original draft, Writing – review & editing. **Rizvee Hassan Prito:** Formal analysis, Validation, Data curation. **Md. Shahadat Anik Sheikh:** Data curation, Writing – original draft, Writing – review & editing. **Md Sawkat Ali:** Supervision, Funding acquisition, Conceptualization. **Mahamudul Hasan:** Supervision, Funding acquisition, Validation. **Maheen Islam:** Supervision, Conceptualization.

## Data Availability

Dataset of Annotated Rice Panicle Image from Bangladesh (Original data) (Mendeley Data)
